# Brief Acoustic Tinnitus Suppression as a Diagnostic Procedure in Clinical Routine: Feasibility and Results

**DOI:** 10.1007/s10162-025-01004-0

**Published:** 2025-09-18

**Authors:** Stefan Schoisswohl, Martin Vizethum, Martin Schecklmann, Andreas Reissmann, Veronika Vielsmeier, Katharina Kerkel, Berthold Langguth

**Affiliations:** 1 Department of Psychiatry and Psychotherapy, University of Regensburg, Regensburg, Germany; 2 Department of Human Sciences, Institute of Psychology, Universitaet der Bundeswehr Muenchen, Neubiberg, Germany; 3 Department of Otorhinolaryngology, University of Regensburg, Regensburg, Germany

## Abstract

Brief acoustic tinnitus suppression (BATS) is a well-known phenomenon among tinnitus patients. Most knowledge about BATS comes from experiments applying filtered, modulated, or customized stimuli in selected patient populations. Testing BATS in clinical routine could provide valuable information for patient subtyping and assistance in treatment decision-making. Here, we investigated the feasibility of BATS tests beyond controlled experimental settings. Seventy individuals with tinnitus (29 female) were tested for BATS using white noise as part of a first consultation visit at the Interdisciplinary Tinnitus Center in Regensburg. The procedure turned out to be feasible under clinical routine conditions. Thirty-five patients (50%) reported some form of tinnitus suppression, with 6 (8.6%) reporting at least 50% reduction and 1 (1.4%) complete absence of their tinnitus percept. The degree of suppression was rated as relevant improvement by most patients. In summary, the integration of BATS assessments was feasible and provided valuable information about the patients’ tinnitus.

**Supplementary Information:**

The online version contains supplementary material available at 10.1007/s10162-025-01004-0.

## Introduction

In a significant proportion of individuals with tinnitus, the tinnitus percept can be temporarily suppressed or decreased following acoustic stimulation with ordinary white noise (WN) or various types of filtered and/or modulated sounds [1]. This phenomenon of brief acoustic tinnitus suppression (BATS) is typically referred to as residual inhibition [2]. The intensity and length of BATS vary from person to person, and in some cases, even increases in tinnitus loudness after sound stimulation are reported [1]. The prevalence of BATS in tinnitus patients differs among studies and ranges between 50 and 90% [3]. For example, Hu et al. [4] could demonstrate that 78% of investigated tinnitus patients experience some level of BATS. In previous experiments, it was shown that tinnitus patients exhibiting BATS report lower levels of tinnitus loudness and tinnitus-related distress as well as lesser chronification grades [4, 5]. These findings support BATS responsiveness as a relevant criterion for tinnitus subtyping. However, knowledge about its prevalence or the influence of sample features mainly comes from experiments under controlled laboratory conditions, often with predefined eligibility criteria and tailored stimulation paradigms. Data about BATS responsiveness from clinical routine settings is rather scarce. Hence, the objective of the present work was to address this gap and evaluate the feasibility and prevalence of BATS by means of WN stimulation in individuals with tinnitus seen in a first consultation visit at the Interdisciplinary Tinnitus Center of Regensburg, Germany.

## Methods

All data were collected and analyzed as part of the framework of the Tinnitus Research Initiative database [6], which was approved by the ethics committee of the University of Regensburg, Germany (08/046). People suffering from tinnitus who attended the tinnitus consultation at the Interdisciplinary Tinnitus Centre in Regensburg, Germany (involving a visit at the Department of Psychiatry and Psychotherapy of the University of Regensburg, Germany), between December 2022 and September 2023 were offered to participate in a facultative additional layer to their consultation visit. The aim of this supplement was to test for BATS susceptibility. In general, this consultation visit is either initiated on people’s own initiative or upon referral from an external expert (e.g., general physician, otolaryngologist, audiologist).

At the beginning of the consultation visit, each patient was fully informed about all study procedures and gave written informed consent. Patients were requested to fill out the German versions of the Tinnitus Sample Case History Questionnaire (TSCH-Q), the Tinnitus Handicap Inventory (THI), the Tinnitus Functional Index (TFI), the Major Depression Inventory (MDI), and Numeric Rating Scales (NRS; 0–10) for tinnitus loudness, tinnitus-related discomfort, annoyance, unpleasantness, and the ability to ignore the tinnitus. Moreover, standard clinical audiometric measurements were performed (Madsen, Midimate, 622D, GN Otometrics, Taustrus, Denmark).

Acoustic stimulation with WN was conducted diotically with an iPad (5th generation; Apple Inc., Cupertino, CA, USA) together with the sound module of the UNITI mobile application [7] and conventional in-ear headphones (MDR-EX15LP; Sony, Tokyo, Japan). Individuals were requested to raise the volume of the WN until their tinnitus was masked. Due to safety reasons and to avoid any risk of noise-induced hearing damage, acoustic stimulation never exceeded a volume level of 85 dB SPL. Only if masking with a maximum volume of 85 dB SPL was possible, patients were stimulated with 1 min of WN and were requested to rate the loudness of their tinnitus on a Visual Analogue Scale from 0% up to 200% in 5% steps (0%, total absence; 100%, usual perceived loudness; 200%, doubled loudness). The decision to use a Visual Analogue Scale anchored from 0 to 200% and track potential tinnitus loudness amplifications was motivated by the fact that a subsample of patients experiences increases in their tinnitus percept following acoustic stimulation [1, 5]. If no loudness decrease was present after 1-min WN stimulation, patients were offered to repeat the procedure with a WN of 3-min length. In case of a tinnitus loudness decrease, participants were further asked whether it would make a positive difference for them if this experienced reduction was permanent, e.g., as a result of treatment (yes/no).

### Statistical Analyses

Besides evaluating the prevalence of BATS (any short-term tinnitus loudness reduction), we aimed for a statistical comparison of sample characteristics between subjects with and without BATS. Numerical data was analyzed by two-sample *t*-tests, whereas categorical data (e.g., association of BATS and sex) was analyzed by either *χ*^2^-tests or Fisher’s exact tests in the case of cell frequencies below five. Analyses were conducted in *SPSS* (vers. 29.0; IBM Corp., Chicago, USA), and figures were created in *R* using *ggplot2* (vers. 4.2.3; R Foundation for Statistical Computing, Vienna, Austria).

## Results

The execution of BATS testing took a maximum of 10 min per subject. Thus, routine procedures were only marginally disrupted. No expensive technical equipment was needed, and its handling was relatively straightforward plus did not require any extensive training for clinical staff or subjects. Seventy individuals with tinnitus (29 female) agreed to participate in the voluntary additional test in the context of their consultation visit and provided the necessary data for the aim of the present work. Participants had a mean age of 53.94 ± 11.60 years and reported perceiving their tinnitus for 114.18 ± 121.88 months on average. On a group level, a moderate tinnitus-related handicap (grade 3) was evident (THI score 50.14 ± 22.15). Detailed sample characteristics are provided in Table 1.

A total number of 35 participants (50%) reported to experience some level of BATS, of whom 25 reported a loudness reduction after 1 min and 10 after 3 min of WN. The average perceived tinnitus loudness during BATS was 70.71 ± 23.61%. Six subjects (8.57%) reported a loudness below 50% (5 after 1 min; 1 after 3 min). One subject (1.43%) reported a (temporary) complete absence of the tinnitus percept after 1 min of WN. Out of 35 participants with BATS, 23 subjects (65.71%) stated that it would make a positive difference for them if the transiently perceived tinnitus reduction would become permanent.

**Table 1:** Sample characteristics

*N* (female)	70 (29)		
Tinnitus side (left/right/bilateral) (1 missing)	12/7/50		
Tinnitus loudness fluctuation (yes/no)	45/25		
	M ± SD	Min	Max
Age (years)	53.94 ± 11.60	24	75
Tinnitus duration (months) (21 missings)	114.18 ± 121.88	5	462
Hearing loss, left (dB) (1 missing)	30.35 ± 14.53	4	101
Hearing loss, right (dB) (1 missing)	28.04 ± 10.46	3	48
Tinnitus frequency, left (Hz) (20 missing)	6912.00 ± 3578.13	250	14.000
Tinnitus frequency, right (Hz) (20 missing)	6922.00 ± 3579.08	250	14.000
THI score (0–100)	50.14 ± 22.15	4	94
TFI score (0–100)	51.94 ± 23.04	1	99
MDI score (0–50) (2 missings)	14.69 ± 11.53	0	42
NRS tinnitus loudness (0–10) (1 missing)	6.62 ± 2.14	1	10
NRS tinnitus discomfort (0–10) (1 missing)	6.87 ± 2.39	1	10
NRS tinnitus annoyance (0–10)	6.99 ± 2.64	0	10
NRS tinnitus ignorability (0–10)	6.93 ± 2.69	0	10
NRS tinnitus unpleasantness (0–10)	6.90 ± 2.57	1	10
Stimulation loudness (dB)	59.28 ± 13.05	35	85

*M* mean, *SD* standard deviation, *THI* Tinnitus Handicap Inventory, *TFI* Tinnitus Functional Index, *MDI* Major Depression Inventory, *NRS* Numeric Rating Scale

**Fig. 1:**
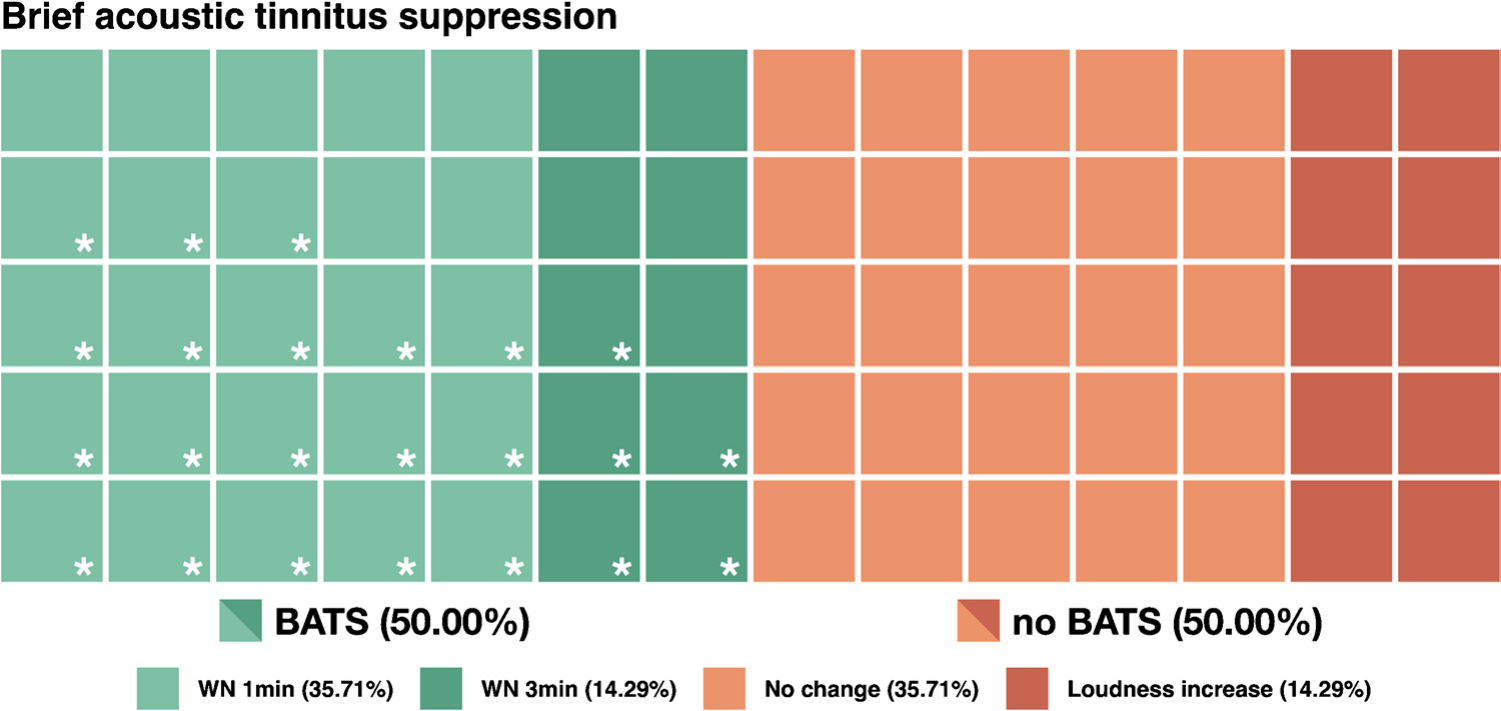
Brief acoustic tinnitus suppression (BATS). Each square represents an individual with tinnitus who took part in the evaluation of BATS (*N* = 70). 35 participants (50%) reported BATS after white noise (WN) stimulation of either 1 (25) or 3 (10) min. White asterisks highlight the 23 participants for which it would make a positive difference if the experienced temporary tinnitus loudness decrease would be permanent. Another 35 participants (50%) did not experience any change in their tinnitus percept after acoustic stimulation (no BATS), whereas 10 of whom report a loudness increase

In another 35 participants (50%), it was not possible to evoke a temporary reduction of the tinnitus percept with 1 or 3 min of WN. Ten subjects (14.29%) experienced an increase in their usually perceived tinnitus loudness. In one subject (1.43%), tinnitus loudness transiently doubled after stimulation. The average tinnitus loudness after WN stimulation in individuals without BATS was 107.57 ± 19.27%. An overview of the quantity of participants with and without BATS is illustrated in Fig. 1. Tinnitus loudness evaluations for individuals with and without BATS can be seen from Figure S1 in the Supplementary Material.

No significant group difference (BATS vs. no BATS) in sample characteristics such as tinnitus duration or loudness was present. No association with sex, tinnitus laterality, or loudness fluctuation with BATS was observed. A detailed overview of sample characteristics and group comparisons can be found in Table S1 in the Supplementary Material.

## Discussion

The aim of the present study was to test the feasibility of brief acoustic tinnitus suppression (BATS) in clinical routine and to collect first results from a naturalistic patient sample.

The integration of BATS assessments into our clinical workflow imposed only marginal time, technical, and personnel requirements and can therefore be considered feasible under clinical routine conditions.

With the present investigation, we can put into perspective a prevalence of 50% for BATS, with six subjects (8.6%) reporting more than 50% reduction of their usually perceived tinnitus loudness and one subject (1.4%) stating complete absence of the tinnitus percept. For 65.7% of these patients, it would make a substantial positive difference if the perceived transiently decreased tinnitus loudness would be permanent. Even if there is currently no tinnitus treatment which can effectively mitigate tinnitus loudness [8], the observed transient tinnitus reduction provides hope that tinnitus loudness reduction of comparable magnitude might be achievable by future treatments, e.g., by permanently activating the inhibitory mechanisms involved in the BATS phenomenon.

The majority of previous studies report higher BATS occurrence, ranging from 50 to 90%, and greater rates of complete tinnitus suppression varying from 3 to 72% [1, 3, 4]. Unlike our findings, these data come from dedicated experiments under laboratory conditions mostly involving selected participants and using a set of different filtered and/or modulated stimuli adapted to a patient’s individual tinnitus characteristics, as these stimuli are supposed to induce superior temporary suppressions. In the present investigation, we used unmodified WN since it can be easily applied in the clinical setting, covers a broad range of frequencies, and is traditionally investigated in this context.

Recently, the analysis of pooled data from several experiments using various stimulus types revealed a prevalence of 47.9% for BATS [9]. In one of these experiments, we could demonstrate that in 26.7% of participants, a suppression of at least 50% can be evoked by applying different types of noise stimuli, whereby in 11.1%, it was feasible by WN [5].

These findings are in a similar range as the prevalence rate (50%) and the number of patients reporting a minimum loudness reduction of 50% (8.6%) observed in the present study using WN. Hence, the application of WN for the investigation of BATS appears to be most appropriate not only from a practical perspective but also in terms of tinnitus loudness suppression quality, as the superiority of customized sounds remains inconclusive.

Notably, one person reported a temporary total absence of the tinnitus percept after stimulation. Cases of complete tinnitus suppression following sound stimulation occur infrequently but can persist for several minutes to several days [3].

Typically, a subset of individuals also experiences some degree of tinnitus loudness increases [5]. In the present study, we can report that in 14.3% of participants, the tinnitus percept increased after 1 min of WN stimulation.

In contrast to previous investigations, we did not observe any differences in demographic factors between participants with and without BATS [4, 5]. This result somewhat questions the relevance of BATS as a criterion for tinnitus subtyping. Future studies should investigate whether the susceptibility to BATS predicts response to sound therapy [1]. Patients with the ability to experience brief suppressions of the tinnitus percept reported superior tinnitus symptom relief following sound treatment [10].

Since we were solely interested in the prevalence of the BATS phenomenon, our approach lacks suppression duration assessments. Future research in the clinical setting should strive for a detailed evaluation of the temporal dynamics of BATS and an identification of suppression consistency predictors (short versus long duration), as this may be important for sound treatment response as well.

Taken together, BATS testing is feasible as a diagnostic procedure in clinical routine. 50% of investigated patients perceived a tinnitus reduction. Future studies should investigate whether susceptibility to BATS might represent a predictor for the response to sound-based treatment approaches.

## Supplementary Information

Below is the link to the electronic supplementary material.

ESM1 (PDF 194 KB)

## Data Availability

The dataset analyzed in the present study is available from the corresponding author upon reasonable request.

## Declarations

### Declaration of AI Use

ChatGPT-4o has been used for AI-assisted copy editing.
