# Supplementary material for: Brief Acoustic Tinnitus Suppression as a Diagnostic Procedure in Clinical Routine: Feasibility and Results
Source: J Assoc Res Otolaryngol. 2025 Sep 18;26(6):635–9. doi: 10.1007/s10162-025-01004-0 (PMC12698921; doi:10.1007/s10162-025-01004-0)
Supplement: Supplementary file 1 — (PDF 194 KB) [file 10162_2025_1004_MOESM1_ESM.pdf]

Supplementary Material - Brief Acoustic Tinnitus Suppression as a Diagnostic Procedure in Clinical Routine: Feasibility and Results

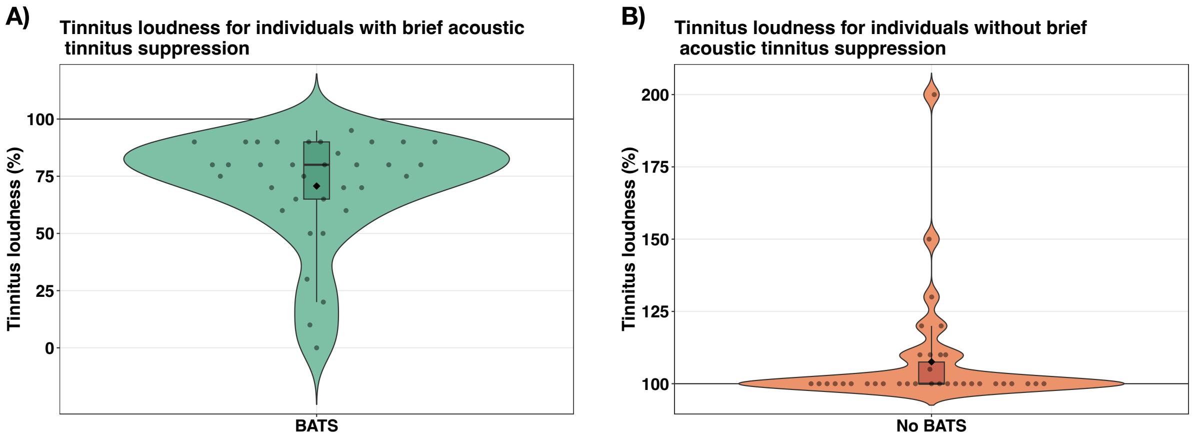

Figure S1. Tinnitus loudness evaluations for individuals with and without brief acoustic tinnitus suppression (BATS). Violin plots with embedded boxplots show the distribution of tinnitus loudness evaluations after the application of WN in individuals **A)** with (n = 35) and **B)** without (n = 35) BATS. A tinnitus loudness rating of 0% represents the total absence of the individuals tinnitus percept, whereas 200% represents a doubling of tinnitus loudness following stimulation. 100% reflects the usual perceived tinnitus loudness (black line). For individuals without BATS the loudness evaluation was averaged over both acoustic stimulation attempts. Mean values are indicated by black-filled diamond markers (BATS: 70.71±23.61%, No BATS: 107.57±19.27%). Grey points indicate tinnitus loudness evaluations per individual subject.

Table S1

Statistical comparison of subjects with and without brief acoustic tinnitus suppression

	BATS	No BATS		*p*
N (female)	35 (11)	35 (18)		*.089*
Tinnitus side (left/right/bilateral) (1 missing)	6/4/24	6/3/26		*.927*
Tinnitus loudness fluctuation (yes/no)	24/11	21/14		*.454*
	**M ± SD**	**M ± SD**	$t_{(df)}$	
Age (years)	53.54 ± 11.73	54.34 ± 11.36	$.29_{(68)}$	*.775*
Tinnitus duration (months) (21 missings)	86.43 ± 105.42	135.00 ± 130.87	$1.39_{(47)}$	*.170*
Hearing loss - left (dB) (1 missing)	29.55 ± 11.73	31.13 ± 16.95	$.45_{(67)}$	*.654*
Hearing loss - right (dB) (1 missing)	28.35 ± 10.88	27.75 ± 10.19	$-.238_{(67)}$	*.813*
Tinnitus frequency – left (Hz) (20 missing)	6535.19 ± 3930.69	7354.35 ± 3142.85	$.804_{(48)}$	*.425*
Tinnitus frequency – right (Hz) (20 missing)	6553.70 ± 3934.14	7354.35 ± 3142.85	$.785_{(48)}$	*.436*
THI score (0-100)	48.74 ± 23.26	51.54 ± 21.22	$.526_{(68)}$	*.601*
TFI score (0-100)	50.18 ± 23.95	53.70 ± 22.30	$.637_{(68)}$	*.526*
MDI score (0-50) (2 missings)	14.15 ± 11.75	15.20 ± 11.47	$.372_{(66)}$	*.711*
NRS tinnitus loudness (0-10) (1 missing)	6.40 ± 2.56	6.85 ± 1.60	$.879_{(67)}$	*.382*
NRS tinnitus discomfort (0-10) (1 missing)	6.66 ± 2.61	7.09 ± 2.17	$.745_{(67)}$	*.459*
NRS tinnitus annoyance (0-10)	6.49 ± 2.96	7.49 ± 2.20	$1.60_{(68)}$	*.114*
NRS tinnitus ignorability (0-10)	6.49 ± 2.93	7.37 ± 2.38	$1.39_{(68)}$	*.170*
NRS tinnitus unpleasantness (0-10)	6.46 ± 2.86	7.34 ± 2.18	$1.46_{(68)}$	*.150*
Stimulation loudness (dB)	59.67 ± 12.71	58.89 ± 13.56	$-.25_{(68)}$	*.805*

BATS = brief acoustic tinnitus suppression, M = mean, SD = standard deviation, THI = Tinnitus Handicap Inventory, TFI = Tinnitus Functional Index, MDI = Major Depression Inventory, NRS = Numeric Rating Scale.
